# Supplementary material for: Deciphering Differential Life Stage Radioinduced Reproductive Decline in Caenorhabditis elegans through Lipid Analysis
Source: Int J Mol Sci. 2021 Sep 24;22(19):10277. doi: 10.3390/ijms221910277 (PMC8508812; doi:10.3390/ijms221910277)
Supplement: Supplementary file 1 [file ijms-22-10277-s001.zip › ijms-1371893-supplementary.pdf]

International Journal of *Molecular Sciences*

MDPI

Deciphering differential life stage radioinduced reproductive decline in *Caenorhabditis elegans* through lipid analysis

Elizabeth Dufourcq-Sekatcheff [1,*], Stephan Cuiné [2], Yonghua Li-Beisson [2], Loïc Quevarec [1], Myriam Richaud [3], Simon Galas[3] and Sandrine Frelon [1,*]

[1] IRSN PSE-ENV Laboratoire de recherche sur les effets des radionucléides sur l'environnement, Cadarache Saint Paul les Durance 13115, France; elizabeth.dufourcq-sekatcheff@irsn.fr; loic.quevarec@irsn.fr
[2] Aix Marseille Univ, CEA, CNRS, Institut de Biosciences et Biotechnologies Aix-Marseille, BIAM, CEA Cadarache, 13108 Saint Paul-Lez-Durance, France ; stephan.cuine@cea.fr ; yonghua.li@cea.fr
[3] IBMM Université de Montpellier, CNRS, ENSCM, Montpellier, France ; myriam.richaud@umontpellier.fr ; simon.galas@umontpellier.fr
* Correspondence: elizabeth.dufourcq-sekatcheff@irsn.fr ; sandrine.frelon@irsn.fr

Exposure Scenario	Cumulated Dose (Gy) (mean ± SD)
SC1	1,10 ± 0,2
SC2	2,41 ± 0,2
SC3	3,23 ± 0,22
OP50	2,92 ± 0,21

Table S1. Cumulated doses for each condition

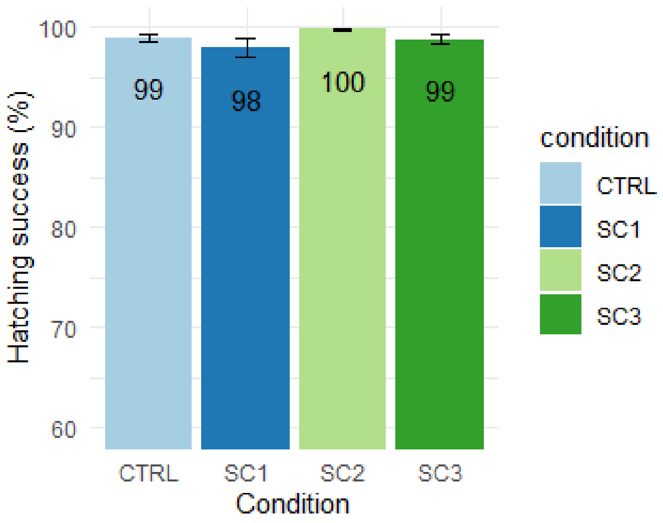

Figure S1. Hatching success (means ± se, n=18-20 for each condition - GLM on total unhatched eggs)

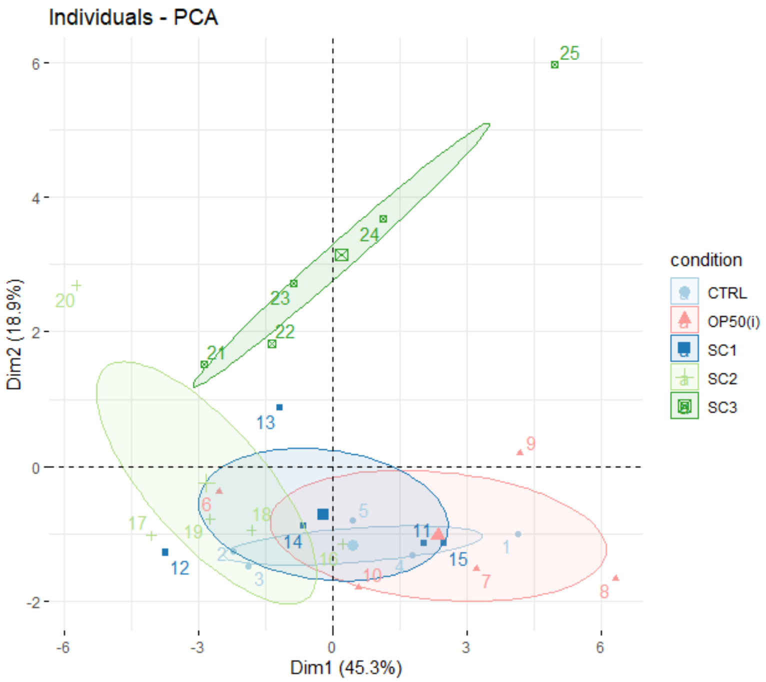

Figure S2. Graph of individuals of the Principal Component Analysis on FAMEs (5 point for each condition corresponding to each replicate (CTRL 1-5, OP50(i) 6-10, SC1 11-15, SC2 16-20, SC3 21-25 and confidence ellipses at 95% for each condition)

FAMEs	Correlation with dose	Dim.1 (%)	Dim.2 (%)
C12:0	0.50*	5,20	6,14
C14:0	0.29	7,36	4,73
C15:0	0.07	8,57	0,16
C16:0	-0.17	5,15	0,00
C16:1(9)	0.53*	0,78	22,83
C16:1	0.59**	0,18	8,33
C17:0iso	0.05	6,62	0,89
C17:0	0.26	5,52	5,52
C17Δ	-0.73**	5,39	10,16
C18:0	0.03	7,54	1,33
C18:1(9)	-0.09	7,93	0,01
C18:1(11)	-0.29	9,28	0,87
C18:2(9-12)	-0.39	9,35	1,75
C18:3	0.28	0,10	8,08
C19:0	-0.50*	0,97	0,12
C18 (3-hydroxy)	-0.60**	6,45	3,25
C19Δ	-0.64**	4,23	12,90
C20:3	-0.18	0,94	0,74

C21:5	-0.36	3,93	7,05
C22:1	0.29	4,55	5,13

Table S2. Spearman correlation between total dose (see Table S1) and FAMEs content ('*' p<0,05;'**' p<0,01) and 'FAMES' variable contribution to PCA axis

Assessment of effects on food behavior and assimilation

(i) Bacterial lawn avoidance assay

At the end of exposure, L4/YA worms from controls, OP50(i) and SC3 were transferred into fresh NGM plates with un-irradiated OP50 lawn. 20 worms were individually deposited onto the NGM plates in triplicates (for a total of 60 worms per condition), in an equidistant manner outside of the bacterial lawn. The number of worm reaching the bacterial lawn was measured during 28h, every hour during the first 8h and from 24 to 28h. Percentage of occupancy was calculated by dividing the number of worms in the bacterial lawn by the total number of worms deposited.

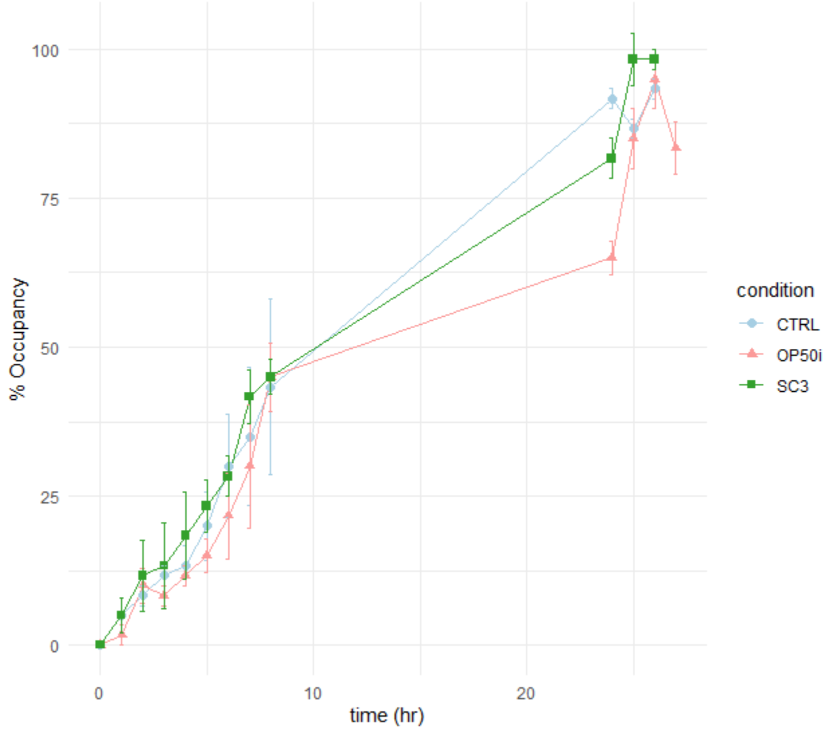

Figure S3. Time course of attraction of nematodes to *E. coli* OP50 lawn for each condition. Error bars indicate SEM.

(ii) Thrashing assay

At the end of exposure, ~20 L4/YA worms per condition were deposited into 12-well NGM plates. Worms were left for a few minutes to get rid of bacteria, then 1mL of M9 was added to the plate onto the worm. Worms were left ~30 seconds to adjust to the liquid medium, then the number of thrashes defined by a single inflection back and forth was counted for 30s.

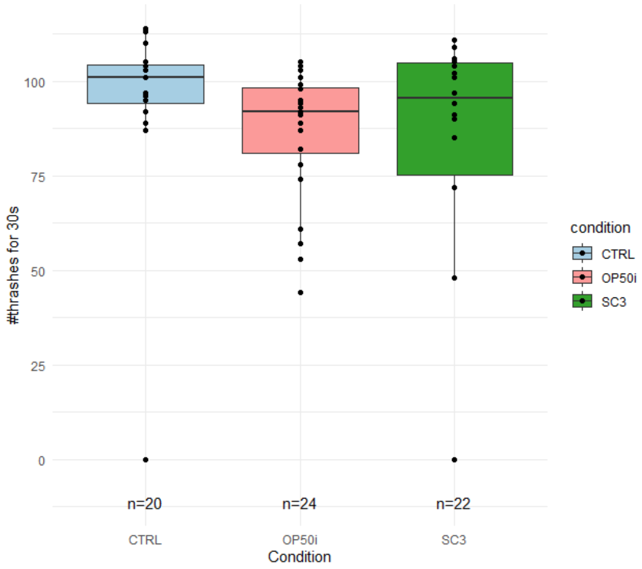

Figure S4. Number of thrashes for 30s for each condition (Kruskal-Wallis)

(iii) Assimilation of OP50

At the end of exposure, worms were rinsed and transferred into NGM plates containing OP50 previously stained with cyanine 5 (Cy5). Worms were left to feed onto the stained bacteria for ~6h then lightly rinsed and mounted onto slides with agar pad and a drop of 25mM NaN_3. Images were obtained with a LSM 780 confocal microscope (Zeiss) using a 20x dry at a resolution of 12 bits, and the red HeNe laser (633 nm) for excitation of Cy5. Whole worm intensity and intensity only inside the digestive tract were measured using the ROI and measure tools in FIJI. Assimilation efficiency was then calculated using the formula explained in Supplementary Data (Figure S6).

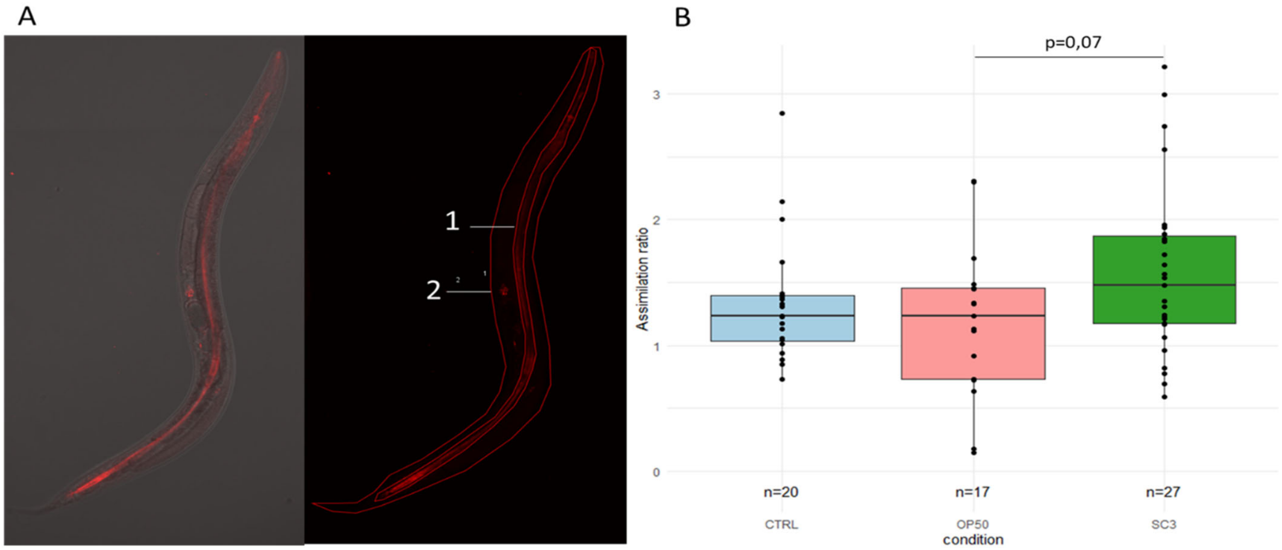

Figure S5. (A) Method for measurement of assimilation rate (Cy5-stained OP50 in the intestine). (B) Assimilation ratio* for each condition (Anova, Tukey post-hoc)

$$* \, Assimilation\ ratio = \frac{(Intensity\ 2 - Intensity\ 1)}{Intensity\ 1}$$

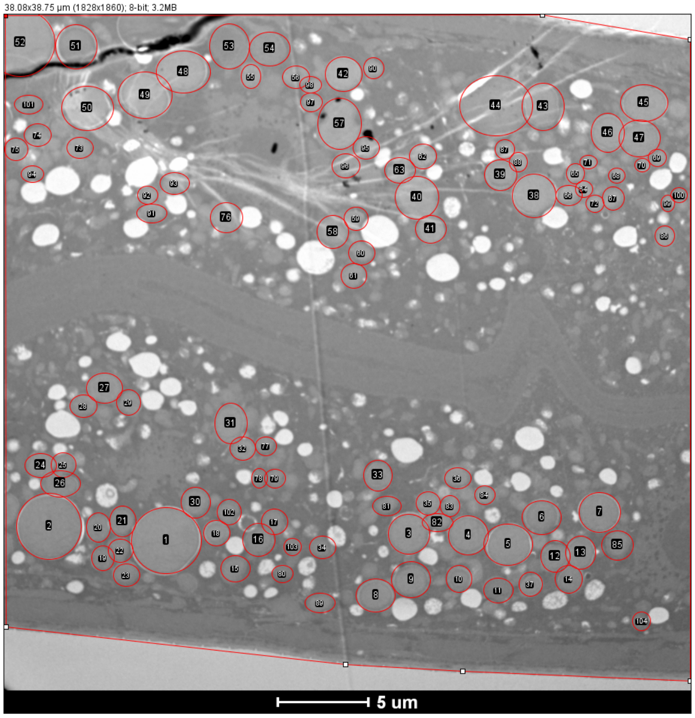

Figure S6. Example of analyzed image (CTRL) for lipid droplet area measurement using FIJI (ROI Manager tool)
